# Case Report: A novel MYBPC3 gene variant in a Chinese patient with hypertrophic cardiomyopathy and apical ventricular aneurysm, with a concurrent novel KCNJ5 gene variant

**DOI:** 10.3389/fcvm.2026.1841777

**Published:** 2026-06-25

**Authors:** Shan Han, Ying-Yi Zhang, Jie Geng

**Affiliations:** Department of Cardiology, Chest Hospital, Tianjin University, Tianjin, China

**Keywords:** apical ventricular aneurysm, case report, hypertrophic cardiomyopathy, KCNJ5 gene variant, MYBPC3 gene variant

## Abstract

Hypertrophic cardiomyopathy (HCM) is an autosomal dominant primary myocardial disease, primarily caused by pathogenic variants in genes encoding sarcomere-associated proteins. Among these, the cardiac myosin-binding protein C (MYBPC3) gene and the MYH7 gene are the two most common causative genes in patients with HCM, accounting for approximately 70% of cases with positive genetic variants. In patients with HCM, left ventricular aneurysm has been reported in fewer than 5% of cases in the absence of coronary artery disease. Variants in the KCNJ5 gene often lead to familial hyperaldosteronism type III and long QT syndrome type 13. Here, we report a rare case of HCM complicated by an apical ventricular aneurysm, accompanied by novel mutations in MYBPC3 and KCNJ5. This rare case, featuring two novel genetic variants, will help expand research on the genotype–phenotype relationship in hypertrophic cardiomyopathy.

## 1 Introduction

Hypertrophic cardiomyopathy (HCM) is clinically defined as a disease state in which morphological abnormalities are confined to the heart. It is characterized predominantly by left ventricular hypertrophy (LVH) in the absence of any other cardiac, systemic, or metabolic condition capable of producing the observed degree of hypertrophy in a given patient. In such cases, either a disease-causing sarcomeric (or sarcomere-related) gene variant is identified, or the genetic etiology remains unresolved. To date, variants in one or more of at least eight genes encoding proteins of the cardiac sarcomere (or sarcomere-related structures) have been implicated in causing LVH—the hallmark feature of HCM. Among patients with HCM and a pathogenic sarcomeric gene variant, the two most commonly involved genes are beta-myosin heavy chain 7 (MYH7) and myosin-binding protein C3 (MYBPC3), which account for the majority of variant-positive cases. Other genes—including TNNI3, TNNT2, TPM1, MYL2, MYL3, and ACTC1—each contribute to a small proportion of patients (1%–5%) (1).

HCM complicated by apical ventricular aneurysm is relatively rare, occurring in approximately 5% of patients with HCM. The presence of such an aneurysm may increase the risk of heart failure, stroke, and ventricular arrhythmias (1). While sarcomeric variants leading to HCM are predominantly missense mutations in most genes, more than half of the pathogenic variants in MYBPC3 are insertions/deletions or splice-site alterations that result in truncated protein expression. In contrast, variants in the KCNJ5 gene are typically associated with familial hyperaldosteronism type III and long QT syndrome type 13. Here, we report a case of a patient with hypertrophic cardiomyopathy and ventricular aneurysm who carries both a novel MYBPC3 gene variant and a novel KCNJ5 gene variant.

## 2 Case presentation

The proband was a 63-year-old woman who presented to the outpatient department in July 2025 with a 7-day history of palpitations. She had been admitted to a local hospital seven days earlier due to sudden-onset palpitations. An electrocardiogram at that time showed atrial fibrillation, which reverted to sinus rhythm after treatment with intravenous amiodarone (total dose: 450 mg). She was diagnosed with arrhythmia and prescribed aspirin and metoprolol. However, she continued to experience intermittent palpitations, leading to her current hospitalization.

She had no history of hypertension, diabetes, hyperlipidemia, or other systemic diseases, and denied smoking, substance use, or alcohol abuse. There was no family history of cardiac disease or sudden cardiac death at a young age. On examination, her body mass index (BMI) was 15.6 kg/m^2^, blood pressure measured 109/73 mmHg, and heart rate was 56 beats per minute. Electrocardiography (EKG) revealed sinus bradycardia, *P*-wave widening, atrial premature beats, and right bundle branch block (Figure 1F). Chest CT scan showed bilateral pleural thickening with adhesions.

**Figure 1:**
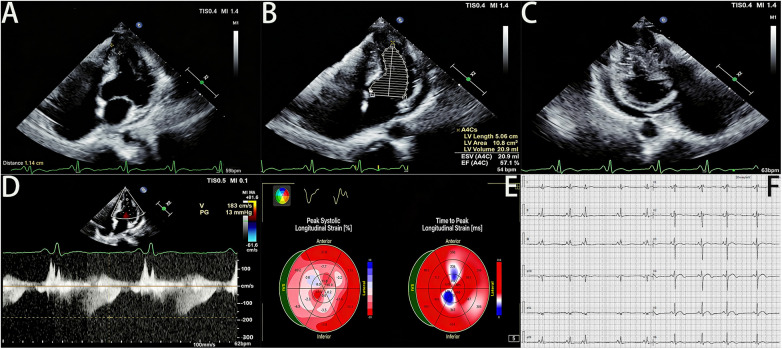
Patient's echocardiography findings. **(A)** Echocardiographic results at the time of hospitalization. **(B)** Relevant echocardiographic parameters. **(C)** Visually evident mid-ventricular obstruction during ventricular systole and an apical ventricular aneurysm. **(D)** Mid-ventricular pressure and flow velocity during ventricular systole. **(E)** Left ventricular global longitudinal strain. **(F)** Electrocardiogram findings at admission.

Echocardiography demonstrated the following: left ventricular end-diastolic diameter (LVEDd) 43 mm, left atrial diameter (LAD) 30 mm, right atrial diameter (RAD) 46 mm, right ventricular end-diastolic diameter (RVEDd) 30 mm, interventricular septal thickness in diastole (IVSd) ranging from 11 to 16 mm, left ventricular posterior wall thickness in diastole (LVPWd) 9–16 mm, pulmonary artery systolic pressure 50 mmHg, and left ventricular ejection fraction (LVEF) 57% (Figures 1A,B). The mid to distal segments of the left ventricular walls showed myocardial thickening, with a maximum thickness of approximately 16 mm. The left ventricular apex exhibited localized thinning of the myocardium (approximately 4–5 mm), with outward bulging and mild paradoxical motion (Figure 1C). No increased systolic flow velocity was observed across the left ventricular outflow tract at rest or after the Valsalva maneuver. The mid-cavity flow velocity was normal at rest and increased to approximately 1.8 m/s after provocation, corresponding to a pressure gradient of 13 mmHg (Figure 1D). A pericardial effusion was also noted, with approximately 8 mm of fluid posterior to the left ventricular wall. The echocardiographic findings were consistent with hypertrophic cardiomyopathy complicated by apical ventricular aneurysm, with consideration of concomitant mid-cavity obstruction.

Left ventricular global longitudinal strain (GLS) is a sensitive measure for detecting regional and global alterations in LV function. In patients with HCM, strain abnormalities predominantly occur in areas of hypertrophy—such as the septum in classic septal hypertrophy or the apex in apical variants. Speckle-tracking analysis of left ventricular myocardial strain in this proband demonstrates reduced longitudinal strain in the mid-to-basal segments of all LV walls and in the basal segment of the interventricular septum, with a global longitudinal strain of approximately −12.1%. Concurrently, speckle tracking revealed no significant apical sparing (the “strawberry sign”), a finding that does not support cardiac amyloidosis (Figure 1E).

To rule out other potential causes of myocardial hypertrophy, additional investigations were performed. Laboratory tests revealed elevated B-type natriuretic peptide (BNP 576.09 pg/mL) and elevated Troponin T (TnT 0.041 ng/mL). Other parameters—including complete blood count, coagulation profile, D-dimer, liver and kidney function, blood glucose, electrolytes, thyroid function, iron studies, autoimmune markers, tumor markers, serum free light chains, serum protein electrophoresis, and immunofixation electrophoresis—were within normal limits. A 24-h Holter monitor recording showed frequent atrial premature beats, short runs of atrial tachycardia, and brief episodes of ventricular tachycardia. Given that coronary artery disease is a common cause of apical ventricular aneurysm, coronary CT angiography was performed, which showed no significant coronary stenosis.

Cardiovascular magnetic resonance (CMR) demonstrated the following: left ventricular end-diastolic volume (EDV) 70 mL, LVEDd 43 mm, LVEF 62%, and cardiac output (CO) 2.73 L/min. The basal interventricular septum measured approximately 12 mm in thickness, the mid-septum about 14 mm, and the apical septum up to 20 mm, accompanied by localized myocardial fibrosis (involving less than 5% of the myocardium). An apical left ventricular aneurysm was also observed (Figures 2A–C). Late gadolinium enhancement images revealed transmural enhancement in the anterior and inferior walls of the apical segment, as well as in the anterior septum of the mid-ventricular segment (Figures 2D–F).

**Figure 2:**
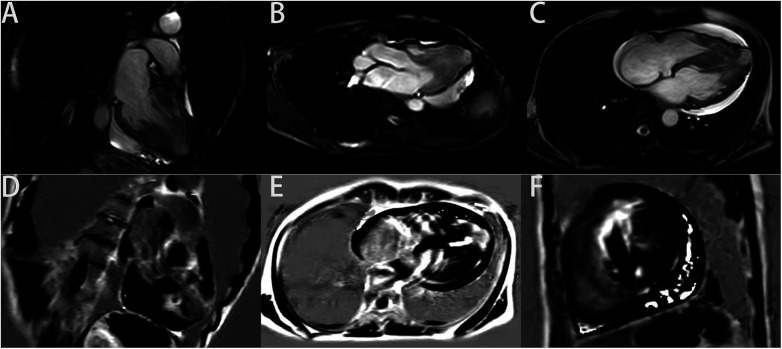
Patient's MRI findings. **(A)** Cardiac MRI, two-chamber view. **(B)** Cardiac MRI, three-chamber view. **(C)** Cardiac MRI, four-chamber view. All three panels show visually evident mid-ventricular obstruction during systole and an apical ventricular aneurysm. **(D)** Late gadolinium enhancement (LGE) image, two-chamber view. **(E)** Late gadolinium enhancement (LGE) image, three-chamber view. The highlighted area on the interventricular septum represents fibrosis. **(F)** Late gadolinium enhancement (LGE) image, short-axis view. The highlighted area on the interventricular septum represents fibrosis.

Based on the diagnostic criteria for hypertrophic cardiomyopathy, the proband in this case had a maximum myocardial thickness of 20 mm (as demonstrated by CMR), and the examination findings did not suggest other causes of myocardial hypertrophy, such as hypertensive heart disease (the patient has no history of hypertension, and the myocardial hypertrophy is non-uniform), Fabry disease (lack of typical extra-cardiac manifestations and negative genetic testing for GLA), or cardiac amyloidosis (excluded by the absence of a “strawberry sign” on imaging, normal serum free light chains, negative serum protein electrophoresis, and negative immunofixation electrophoresis). Therefore, the diagnosis of hypertrophic cardiomyopathy was established.

Following ethical approval and acquisition of written informed consent, targeted region capture combined with next-generation high-throughput sequencing was performed to analyze all exons and flanking regions (Testing platform: Illumina NovaSeq 6,000 System; Panel scope: The test is whole exome sequencing, not panel sequencing, covering the exonic and flanking regions of over 20,000 genes; Sequencing depth: Average sequencing depth of 117.87×). All rare and known variants were validated by Sanger sequencing. Cascade screening via Sanger sequencing was subsequently carried out in all available family members.

Genetic analysis identified a novel heterozygous variant NM_000256.3 (MYBPC3):c.278delA (p.Lys93ArgfsTer3) in exon 2 of the MYBPC3 gene, as well as a novel heterozygous variant NM_000256.3 (KCNJ5):c.464G > A (p.Arg155Gln) in exon 2 of the KCNJ5 gene in the proband. Unfortunately, both of the proband's parents are deceased—one due to cancer and the other of natural causes at age 92—with no history of cardiovascular disease reported in either. The proband has no siblings. Her only first-degree relative is her son, who was found to carry only the KCNJ5 c.464G > A variant and did not inherit the MYBPC3 c.278delA variant (Figures 3A,B). His echocardiogram was within normal limits, showing no evidence of myocardial hypertrophy.

**Figure 3:**
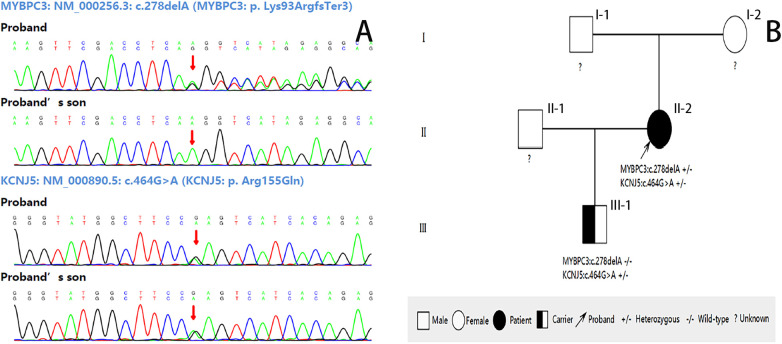
Sanger sequencing results and the pedigree of the family. **(A)** Sanger sequencing results showing the MYBPC3 gene variant NM_000256.3:c.278delA (p.Lys93ArgfsTer3) and the KCNJ5 gene variant NM_000256.3:c.464G > A (p.Arg155Gln). **(B)** Pedigree of the family showing the genotypes of the proband and affected relatives. Squares indicate males, and circles indicate females.

Given the proband's relatively low heart rate and blood pressure, a low-dose pharmacotherapy regimen was initiated. After 13 days of treatment with dapagliflozin, metoprolol, rivaroxaban, and recombinant human brain natriuretic peptide, her clinical status improved. She was discharged on dapagliflozin, metoprolol, and rivaroxaban. At the one-week follow-up, the patient reported persistent palpitations, prompting the addition of mexiletine to her therapeutic regimen. Implantation of an Implantable Cardioverter Defibrillator (ICD) was recommended; however, the patient declined this intervention due to financial constraints. During the subsequent 3-month follow-up, the patient showed good adherence, taking medication regularly. Occasional palpitations occurred, but no serious arrhythmic events were observed, and there were no significant adverse effects. (The case timeline table 1 is provided in the Supplementary Materials).

## 3 Discussion

Genetic testing in patients with HCM provides several key benefits: identifying the underlying genetic cause, distinguishing phenocopies, enabling cascade screening within families, facilitating genetic counseling, and providing access to emerging therapies. For example, active gene therapy trials are currently underway targeting genes such as MYBPC3 and LAMP2 (2).

The MYBPC3 gene encodes the cardiac isoform of myosin-binding protein C (cMyBP-C). This gene comprises a 3.7 kb DNA sequence containing 34 coding elements that give rise to 35 exons (3). The primary function of cMyBP-C is to precisely regulate cross-bridge cycling in cardiomyocytes through phosphorylation and interactions with other regulatory factors, thereby contributing to the assembly of myosin heavy chains into the sarcomere. This regulatory mechanism is essential for proper cardiac muscle contraction and relaxation. In addition, cMyBP-C modulates the calcium sensitivity of the thin filament, which directly influences myocardial contractile performance (3).

Of the known MYBPC3 mutations, over 60% are truncating mutations, encompassing nonsense mutations, insertions or deletions, and splicing or branch point mutations. Notably, MYBPC3 exhibits tolerance to both missense and loss-of-function (LoF) mutations (3). Truncating mutations frequently activate nonsense-mediated mRNA decay (NMD) and the ubiquitin–proteasome system (UPS), resulting in MYBPC3 protein haploinsufficiency (4). This haploinsufficiency leads to myosin dysfunction, which represents a central pathophysiological mechanism in HCM. Specifically, MYBPC3 truncation causes destabilization of myosin conformations, producing an altered ratio of disordered relaxation to super-relaxation (DRX/SRX) states. This imbalance promotes hypercontractility, impairs diastolic relaxation, and increases energy consumption.

The proband was found to carry a heterozygous variant in the MYBPC3 gene, designated as c.278delA (MYBPC3:p.Lys93ArgfsTer3). Interrogation of population frequency databases confirmed that this variant is rare, with no reported occurrences in the 1,000 Genomes Project, ESP6500, or ExAC databases. It was also absent from the Besuper local population database, which includes both cardiomyopathy patients and control subjects. This single-base deletion results in the substitution of the positively charged polar residue lysine at position 93 with arginine, another positively charged polar residue, and causes a frameshift during translation. A premature stop codon is introduced at the second subsequent amino acid position, which is predicted to NMD.

No records of this specific variant were identified in the ClinVar or HGMD databases. However, downstream frameshift or nonsense variants at the same locus have been consistently reported in ClinVar and classified as pathogenic or likely pathogenic for hypertrophic cardiomyopathy and related cardiac conditions.

According to the American College of Medical Genetics and Genomics (ACMG) guidelines (5), this variant is classified as ‘Likely Pathogenic’ for HCM, as it meets the following criteria: PVS1 (predicted null variant) and PM2 (absent from population controls). Unfortunately, both parents of the proband have passed away, and the only remaining direct family member is her son. Therefore, we were only able to evaluate one family member, and a valid familial segregation analysis could not be performed. Additionally, functional validation is lacking. Additional evidence may support its future reclassification to ‘Pathogenic’.

In patients with HCM, left ventricular (LV) aneurysm has been reported in less than 5% of cases in the absence of coronary artery disease. Mid-ventricular obstruction (MVO) appears to play a critical role in LV aneurysm formation. Research suggests that MVO significantly elevates myofiber stress at the LV apex, potentially initiating apical aneurysm development directly (6). Furthermore, in the setting of MVO, increased apical stress along with underlying small vessel disease contributes to myocardial injury at the LV apex—consistent with the Fourth Universal Definition of Myocardial Infarction, i.e., an elevation of cardiac troponin above the 99th percentile upper reference limit, as demonstrated in the present case.

In patients with MVO and apical aneurysms, elevated mid-LV pressure gradients may not be observed either at rest or during the Valsalva maneuver. Indeed, MVO—resulting from complete systolic apposition of the LV walls—often occurs in the absence of high-velocity flow. This phenomenon is attributed to near-complete or total cessation of blood flow due to apical akinesia and cavity emptying, which further reduces forward flow through the severely narrowed midventricular segment. Consequently, even when two-dimensional echocardiography suggests visually severe obstruction, the absence of measurable flow results in low recorded velocities. This discrepancy can lead to diagnostic confusion and may result in the misclassification of patients as non-obstructive, despite echocardiographic evidence of complete cavity emptying (6).

Our case illustrates a similar scenario: echocardiography clearly demonstrated visually apparent midventricular obstruction during systole, yet the corresponding pressure gradient measured only 13 mmHg (Figures 1C,D). These observations raise the question of whether invasive assessment is warranted, particularly in symptomatic patients with such anatomical and functional abnormalities. According to the American Heart Association (AHA) guidelines, echocardiography remains the gold standard for noninvasive evaluation of obstruction, and there is currently no established indication for routine invasive hemodynamic assessment in patients with obstructive HCM (7). Given the patient's low body weight and hypotension, invasive evaluation was deferred. Should the patient's clinical status improve with treatment, invasive catheterization may be reconsidered following careful risk–benefit assessment.

LV aneurysm in HCM has been associated with an elevated risk of adverse cardiovascular outcomes (6), specifically an increased incidence of thromboembolic events and sudden cardiac death (SCD). Therefore, LV aneurysm should be incorporated into SCD risk stratification algorithms, and patients with HCM who have an LV aneurysm should be assessed for anticoagulation therapy. In adult HCM patients with one or more major risk factors for SCD, it is reasonable to recommend an ICD. Although ICD implantation was advised for the proband, it was declined due to financial constraints. Rivaroxaban anticoagulation therapy has since been initiated.

Since most MYBPC3 mutations lead to cMyBP-C haploinsufficiency, a direct therapeutic strategy for MYBPC3-related HCM involves introducing wild-type MYBPC3 cDNA into cardiomyocytes. This approach is anticipated to prevent haploinsufficiency without introducing toxicity, as excess sarcomeric proteins are rapidly degraded by the ubiquitin–proteasome system (UPS), which serves as a central regulator for maintaining the stoichiometry of the sarcomeric complex (8). Studies in mouse models and hiPSC-derived cardiomyocytes have demonstrated successful gene replacement using adeno-associated virus (AAV)–mediated delivery of functional MYBPC3, which effectively counteracts protein deficiency and prevents the accumulation of nonsense mRNAs. By employing AAV vectors with high cardiac tropism and cardiomyocyte-specific promoters, any type of MYBPC3 mutation could theoretically be corrected. Given these advances, it is hoped that this proband will be able to benefit from more innovative and effective treatment options in the future.

The KCNJ5 gene is located on the long arm of chromosome 11 (locus 11q24.3) and encodes the potassium channel Kir3.4 (9). It encodes an integral membrane protein and is one of seven subfamilies of inward rectifier potassium channel proteins (referred to as potassium channel subfamily J). The encoded protein is a subunit of the potassium channel and assembles as a homotetramer. It is regulated by G proteins and plays a key role in modulating potassium ion influx. In general, heterozygous variants in KCNJ5 cause familial hyperaldosteronism type III and long QT syndrome type 13. Recent studies have suggested that variants in KCNJ5 are also implicated in Andersen–Tawil syndrome, which is characterized by periodic paralysis and distinctive electrocardiographic features (10).

The proband carried a heterozygous missense variant, c.464G > A (p.Arg155Gln), in the KCNJ5 gene. Querying population frequency databases confirmed this to be a rare variant (1,000 Genomes: absent; ESP6500: absent; ExAC: 8.236e–06). This variant was not detected in individuals with arrhythmias or hypertension, nor in the control population from the local Basenhall database. Cross-prediction using multiple bioinformatics tools (including SIFT and Polyphen-2, among others) consistently indicated deleterious effects (SIFT: “D”; Polyphen-2: “D”; MutationTaster_pred: “D”; VEST4 score: 0.953; REVEL score: 0.934; other tools: 7 D/1 H), suggesting that the resulting amino acid substitution may impact protein function. The change involves a substitution from a positively charged polar arginine to an uncharged polar glutamine. Database queries further revealed that this amino acid position is highly conserved among vertebrates. No records of this specific variant were found in ClinVar or HGMD. However, nearby missense variants, such as c.451G > A (p.Gly151Arg), c.452G > A (p.Gly151Glu), c.470T > G (p.Ile157Ser), and c.473C > G (p.Thr158Arg), have been repeatedly documented in ClinVar as pathogenic or likely pathogenic mutations associated with long QT syndrome or aldosteronism.

According to the American College of Medical Genetics and Genomics (ACMG) guidelines (5), this variant is classified as a variant of uncertain significance (VUS) with moderate evidence of pathogenicity, based on the following criteria: PM2 (absent from population controls) and PP3 (multiple lines of computational evidence support a deleterious effect on the gene or gene product). Currently, familial segregation data and functional studies are lacking to further substantiate its pathogenicity.

While myocardial hypertrophy and related structural damage remain the primary explanations for QTc prolongation in HCM, other mechanisms—such as mutations in long QT syndrome (LQTS) genes—may also contribute. However, one study suggested that variants in certain genes, including KCNJ5, play only a minor role in HCM patients with prolonged QTc and proposed that alternative mechanisms should be considered to explain the electrocardiographic abnormalities observed in these individuals (11). Given that the proband has no history of hypertension and clinical examinations revealed no evidence of conditions associated with familial hyperaldosteronism type III—along with the absence of QT interval prolongation or any manifestations of Andersen–Tawil syndrome (e.g., periodic paralysis) on electrocardiogram—the proband's arrhythmia is more likely attributable to the myocardial structural changes associated with hypertrophic cardiomyopathy. Meanwhile, it is notable that although the proband's son also carries this KCNJ5 variant, no related disease phenotypes have been observed either. Therefore, this gene variant does not appear to have resulted in a clinical phenotype in the present case; it may be an incidental finding or, alternatively, act as a ‘modifier’ rather than a primary causative gene in the context of the proband's arrhythmia. Nonetheless, long-term follow-up with regular electrocardiographic monitoring remains warranted.

## 4 Conclusion

This case report describes two newly identified genetic variants, including a likely pathogenic variant, MYBPC3 c.278delA, associated with HCM. Our findings contribute to broadening the understanding of the genetic landscape underlying HCM pathogenesis and enrich the spectrum of MYBPC3-associated variants documented in both Chinese and global databases. Several limitations should be acknowledged: (1) the absence of functional studies to characterize the molecular consequences of these variants, (2) the lack of mRNA-level validation of these variants, and (3) the inherent limitations inherent to a single-case analysis. These findings highlight the importance of comprehensive genetic testing in patients with HCM, while also underscoring the need for further research to elucidate the pathogenic mechanisms of these rare variants.

## 5 Patient perspective

I always thought I was healthy, experiencing no discomfort whatsoever in my daily life or during exercise. I was admitted to the hospital due to palpitations, and at first, I thought it was just a minor issue. When the doctor told me that I had hypertrophic cardiomyopathy and that it was likely caused by a genetic problem, I was completely stunned. But what scared me the most was the doctor's suggestion that my son should also be tested. I couldn't bear to imagine what I would do if my family members were found to have problems because of me. That sense of guilt was more tormenting than the disease itself.

After my symptoms improved with medication, my panic subsided somewhat. A few weeks later, when I learned that my son did not carry the same MYBPC3 variant and that his echocardiogram showed no significant abnormalities, I breathed a sigh of relief. However, since he does carry a KCNJ5 variant, I will ensure he monitors his ECG and undergoes regular check-ups.

I now feel that although this disease has imposed limitations on me, it has also made our whole family more attentive to our health and more appreciative of one another. I hope that by sharing my story, I can help others understand that with scientific management and regular follow-up after a hypertrophic cardiomyopathy diagnosis, we can live and work just like everyone else. I am also particularly hopeful for medical progress; perhaps one day, a targeted therapy for my specific genetic variant will emerge, allowing us to face the future with greater peace of mind.

## Data Availability

The datasets presented in this study can be found in online repositories. The names of the repository/repositories and accession number(s) can be found in the article/Supplementary Material.
